# Endosomal pH-Responsive Fe-Based Hyaluronate Nanoparticles for Doxorubicin Delivery

**DOI:** 10.3390/molecules26123547

**Published:** 2021-06-10

**Authors:** Yangmun Bae, Yoonyoung Kim, Eun Seong Lee

**Affiliations:** 1 Department of Biotechnology, The Catholic University of Korea, 43 Jibong-ro, Bucheon-si 14662, Gyeonggi-do, Korea; beebe1@naver.com (Y.B.); rladbsdud727@naver.com (Y.K.); 2 Department of Biomedical-Chemical Engineering, The Catholic University of Korea, 43 Jibong-ro, Bucheon-si 14662, Gyeonggi-do, Korea

**Keywords:** Fe-based nanoparticles, endosomal pH-responsive hyaluronate, CD44 receptor-mediated endocytosis, tumor therapy

## Abstract

In this study, we report pH-responsive metal-based biopolymer nanoparticles (NPs) for tumor-specific chemotherapy. Here, aminated hyaluronic acid (aHA) coupled with 2,3-dimethylmaleic anhydride (DMA, as a pH-responsive moiety) (aHA-DMA) was electrostatically complexed with ferrous chloride tetrahydrate (FeCl_2_/4H_2_O, as a chelating metal) and doxorubicin (DOX, as an antitumor drug model), producing DOX-loaded Fe-based hyaluronate nanoparticles (DOX@aHA-DMA/Fe NPs). Importantly, the DOX@aHA-DMA/Fe NPs improved tumor cellular uptake due to HA-mediated endocytosis for tumor cells overexpressing CD44 receptors. As a result, the average fluorescent DOX intensity observed in MDA-MB-231 cells (with CD44 receptors) was ~7.9 × 10^2^ (DOX@HA/Fe NPs, without DMA), ~8.1 × 10^2^ (DOX@aHA-DMA_0.36_/Fe NPs), and ~9.3 × 10^2^ (DOX@aHA-DMA_0.60_/Fe NPs). Furthermore, the DOX@aHA-DMA/Fe NPs were destabilized due to ionic repulsion between Fe^2+^ and DMA-detached aHA (i.e., positively charged free aHA) in the acidic environment of tumor cells. This event accelerated the release of DOX from the destabilized NPs. Our results suggest that these NPs can be promising tumor-targeting drug carriers responding to acidic endosomal pH.

## 1. Introduction

Recently, the biological or physical properties of metal molecules have inspired the development of various bioactive nanoparticles (e.g., bacteria-killing nanoparticles and tumor-targeting nanoparticles) [1,2] and the development of simple biomimetic particles. In particular, metal-containing biopolymer nanocomposites have been actively designed for engineering multifunctional drug carriers with specific biological functions and tunable hyperstructures [1,2,3]. For example, the complexation of Fe and biopolymers is an effective and simple approach to fabricate biocompatible nanosized drug carriers in that Fe is an element present in living organisms and can be used as a chelating agent for negatively charged biopolymers [4,5,6,7]. Moreover, Fe-based nanocomposites are easy to control in terms of their composition, shape, size, and surface characteristics as a result of different mixing ratios and biopolymer types [4,5,6,7]. It is also interesting to note that the combination of biopolymers reactive to specific stimuli (light, temperature, pH, etc.) and Fe can contribute to the development of stimuli-responsive drug delivery systems [4,5,6,7,8,9].

**Figure 1:**
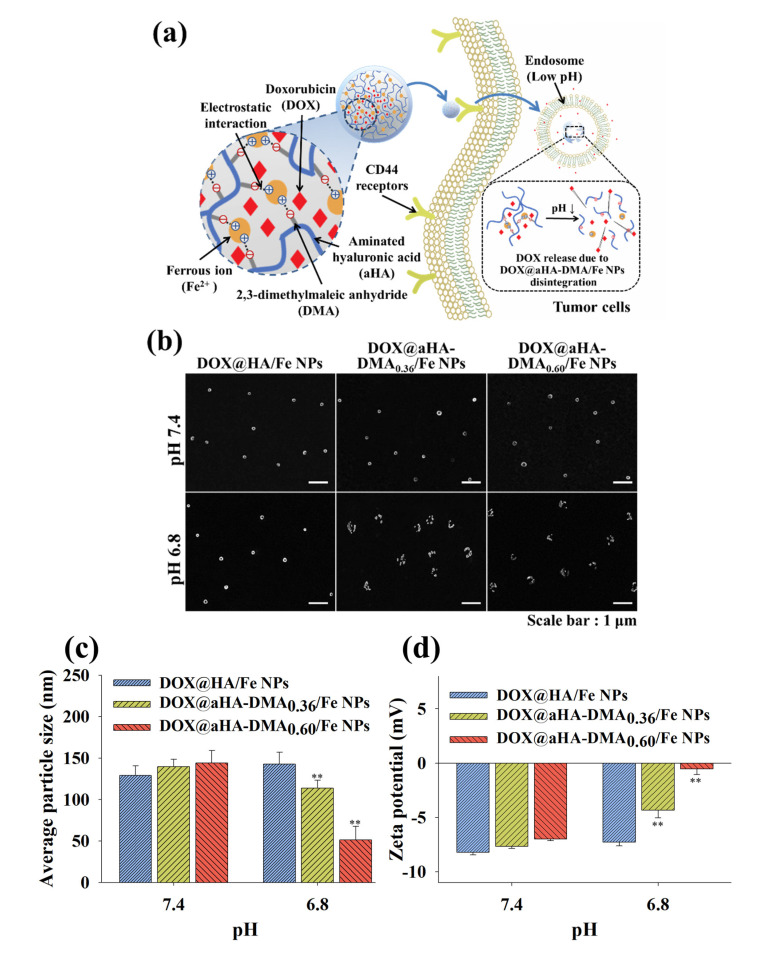
(**a**) Schematic illustration of DOX@aHA-DMA/Fe NPs. (**b**) FE-SEM images of DOX@HA/Fe NPs, DOX@aHA-DMA_0.36_/Fe NPs, and DOX@aHA-DMA_0.60_/Fe NPs at pH 7.4 or 6.8. (**c**) Average particle size and (**d**) zeta potential values of DOX@HA/Fe NPs, DOX@aHA-DMA_0.36_/Fe NPs, and DOX@aHA-DMA_0.60_/Fe NPs at pH 7.4 or 6.8 (*n* = 3, as multiple experiments; ** *p* < 0.01 compared to DOX@HA/Fe NPs).

In this study, we synthesized the aminated hyaluronic acid (aHA) coupled with 2,3-dimethylmaleic anhydride (aHA-DMA), which can target tumor cells with CD44 receptors and is responsive to acidic endosomal pH [10,11,12,13]. Furthermore, we could prepare acidic pH-responsive Fe-based hyaluronate nanoparticles using aHA-DMA, ferrous chloride tetrahydrate (FeCl_2_/4H_2_O) [5,9,12], and doxorubicin (DOX, as an antitumor drug model). It is important that the DMA moiety present in aHA-DMA can be detached from aHA resulting from hydrolysis of DMA at a weakly acidic pH [10,12,14], resulting in the production of positively charged aHA. We expect that our NPs have low cytotoxicity because of the use of biodegradable/biocompatible components (HA and Fe) [12,13,15,16] and exhibit fascinating biological/physicochemical functionality in tumor environments as a result of HA-mediated specific binding to the CD44 receptors of tumor cells and DMA-mediated reactivity to acidic endosomal pH [11,14,15,17,18,19,20,21], resulting in improved antitumor activity (Figure 1a). In this study, we focused not only on the development of Fe-based NPs that have a specific binding ability [11,15] to tumor cells and reactivity to acidic endosomal pH but also on the analysis of the physicochemical properties of NPs.

## 2. Materials and Methods

### 2.1. Materials

Hyaluronic acid (HA, M_w_ = 4.8 kDa), adipic acid dihydrazide (ADH), *N*,*N*′-dicyclohexylcarbodiimide (DCC), *N*-hydroxysuccinimide (NHS), dimethyl sulfoxide (DMSO), triethylamine (TEA), 2,3-dimethylmaleic anhydride (DMA), sodium hydroxide (NaOH), ferrous chloride tetrahydrate (FeCl_2_·4H_2_O), doxorubicin hydrochloride (DOX), formaldehyde, and triton X-100 were purchased from Sigma-Aldrich (St. Louis, MO, USA). Phosphate buffered saline (PBS), Roswell Park Memorial Institute-1640 (RPMI-1640), fetal bovine serum (FBS), penicillin, streptomycin, trypsin, and ethylene diaminetetraacetic acid (EDTA) were purchased from Welgene Inc. (Seoul, Korea). Cell Counting Kit-8 (CCK-8) was purchased from Dojindo Molecular Technologies Inc. (Rockville, MD, USA).

### 2.2. Synthesis of DOX-Loaded Fe-Based HA NPs

HA (300 mg) was preactivated (i.e., aminated) with ADH (644 mg), DCC (770 mg), and NHS (434 mg) in DMSO (15 mL) containing TEA (1 mL) at 25 °C for 4 days. The resulting solution was dialyzed (Spectra/Por^®^ MWCO 3.5 kDa) against fresh DMSO at 25 °C for 3 days and deionized water for 3 days to remove the unreacted chemicals [22]. The aminated HA powder (aHA, 100 mg) obtained through freeze-drying for 2 days was reacted with DMA (174 mg or 87 mg) in 0.1 M NaOH aqueous solution (10 mL, adjusted to pH 9.0 using 0.1 M HCl) for 3 days [10]. This solution was dialyzed using a preswollen dialysis membrane (Spectra/Por^®^ MWCO 3.5 kDa; Spectrum Lab., Rancho Dominguez, CA, USA) against PBS (150 mM, pH 7.4) at 25 °C for 3 days and then lyophilized, producing aHA-DMA_0_._60_ or aHA-DMA_0.36_; the numerals indicate the number of moles of DMA conjugated to 1 repeating unit of aHA (Figure S1).

Next, aHA-DMA_0.60_ (200 mg) or aHA-DMA_0.36_ (200 mg) was mixed with FeCl_2_·4H_2_O (47 mg) and DOX (40 mg) in 0.1 M NaOH aqueous solution (10 mL). The resulting solution was dialyzed using a preswollen dialysis membrane (Spectra/Por^®^ MWCO 3.5 kDa) against PBS (150 mM, pH 7.4) for 3 days and then lyophilized [23,24,25], producing DOX-loaded Fe-based aHA-DMA NPs (DOX@aHA-DMA_0.60_/Fe NPs and DOX@aHA-DMA_0.36_/Fe NPs). In addition, HA (200 mg) was mixed with FeCl_2_·4H_2_O (47 mg) and DOX (40 mg) in 0.1 M NaOH aqueous solution (10 mL) for 3 days to prepare a pH-nonresponsive control group (DOX@HA/Fe NPs). The amount of DOX encapsulated in the NPs was calculated after measuring the fluorescence intensity (*λ_ex_* of 470 nm and *λ_em_* of 592 nm) of DOX remaining in the supernatant (obtained after the centrifugation of solution at 100,000 rpm for 15 min at 4 °C) using a fluorescence spectrophotometer (RF-5301PC, Shimadzu, Kyoto, Japan) [17,18]. The loading efficiency (%) of DOX in the NPs was calculated as the weight percentage of DOX in the NPs relative to the initial feeding amount of DOX. The loading content (%) of DOX in the NPs was calculated as the weight percentage of DOX encapsulated in the NPs [17,18].

### 2.3. Characterization of Fe-Based HA NPs

The surface morphology and particle size of the NPs were monitored using field emission scanning electron microscopy (FE-SEM, Hitach S-400, Nagano, Japan) [12,26]. Before testing, the NPs were stabilized in PBS (150 mM, pH 7.4 or 6.8) at 25 °C for 4 h and then lyophilized. The average particle size and zeta potentials of the NPs (0.1 mg/mL) in PBS (150 mM, pH 7.4 or 6.8) were measured using a Zetasizer 3000 instrument (Malvern Instruments, Malvern, UK) [27,28]. Prior to the experiments, the NPs were stabilized in PBS (150 mM, pH 7.4 or 6.8) at 25 °C for 24 h. In addition, the concentration of Fe in the NPs was measured using an inductively coupled plasma mass spectrometer (ICP-MS, Thermo Scientific Inc., Waltham, MA, USA) [29].

### 2.4. In Vitro DOX Release Behavior

To confirm the pH-dependent DOX release profiles of the NPs at pH 7.4 (i.e., normal body pH) or 6.8 (i.e., acidic endosomal pH), each NP (equivalent to DOX 10 μg/mL) in 1 mL of PBS (150 mM, pH 7.4 or 6.8) was added to a dialysis membrane tube (Spectra/Por^®^ MWCO 50 kDa) and immersed in 15 mL of fresh PBS (150 mM, pH 7.4 or 6.8) [17,18,20,30]. The preswollen membrane tubes were placed in a water bath shaking incubator (100 rpm) at 37 °C. The aqueous solution outside of the dialysis tubes was extracted and changed to a fresh PBS at each time point [17,18,20,30]. The amount of DOX present in the extracted solution was determined by measuring the DOX fluorescence intensity (*λ_ex_* of 470 nm, *λ_em_* of 592 nm) using a fluorescence spectrophotometer (RF-5301, Shimadzu, Kyoto, Japan) [17,18].

### 2.5. Cell Culture

Human breast carcinoma MDA-MB-231 cells and human liver carcinoma Huh7 cells were purchased from the Korea Cell Line Bank (Seoul, Korea) and cultured in RPMI-1640 medium supplemented with 10% FBS and 1% penicillin-streptomycin. The cells were maintained in a humidified incubator with a 5% CO_2_ atmosphere at 37 °C and then harvested via trypsinization using a trypsin/EDTA solution (0.25% (wt./vol.)/0.03% (wt./vol.)). The collected cells (1 × 10^6^ cells/mL) were seeded into a 96-well culture plate and cultured in RPMI-1640 medium for 24 h [11,18,19,20,31,32].

### 2.6. In Vitro Cellular Uptake Experiments

The NPs (equivalent to DOX 10 μg/mL) or free DOX (10 μg/mL) suspended in an RPMI-1640 medium (pH 7.4 or 6.8) were incubated with MDA-MB-231 cells (CD44 receptor-positive cells) and Huh7 cells (CD44 receptor-negative cells) at 37 °C for 4 h. The treated cells were washed three times with fresh PBS (pH 7.4). The DOX fluorescence intensity (*λ_ex_* of 470 nm and *λ_em_* of 592 nm) of the treated cells was analyzed using a flow cytometer (FACS Calibur, Becton Dickinson, Franklin Lakes, NJ, USA) [11,17,31].

To visualize the cellular uptake of DOX, MDA-MB-231 cells and Huh7 cells treated with NPs (equivalent to DOX 10 μg/mL) at pH 7.4 or 6.8 for 4 h were fixed using a 3.7% (wt./vol.) formaldehyde solution and monitored using a visible and near-infrared (VNIR) hyperspectral camera (CytoViva, Auburn, AL, USA). The scanned area of the treated cells was merged with the collected spectral data to visualize the cellular uptake of fluorescent DOX in the cells [11,17].

### 2.7. In Vitro Cytotoxicity

The cells were incubated with NPs (equivalent to DOX 10 μg/mL) or free DOX (10 μg/mL) suspended in an RPMI-1640 medium (pH 7.4 or 6.8) at 37 °C for 4 h and then washed three times with fresh PBS (pH 7.4). The CCK-8 assay was used to evaluate the cell viability of cells treated with the NPs. In addition, the cells were incubated at 37 °C for 24 h with the NPs (1–200 μg/mL, without DOX) at pH 7.4 to evaluate the original toxicity of NPs [11,17].

### 2.8. Hemolysis Test

A hemolysis test was conducted using red blood cells (RBCs) collected from BALB/c mice (7-week-old female) to determine the endosomolytic activity of the NPs. The RBC solution (10^6^ cells/mL, pH 7.4–6.0) was incubated with the NPs (30 µg/mL, without DOX) at 37 °C for 1 h. The solutions were centrifuged at 1500 rpm for 10 min at 4 °C, and the supernatant was collected. The light absorbance (LA) value of the supernatant was measured using a spectrophotometer at a wavelength of 541 nm. The 0% LA value (as a negative control) was acquired from a PBS-treated intact RBC solution and the 100% LA value (as a positive control) was obtained from a completely-lysed RBC solution using 2 wt.% Triton X-100. The hemolysis (%) of NPs was determined as the LA of the RBC solution treated with NPs against the control LA value [11,12,17,18].

### 2.9. Local Healing Assay

The culture dish containing MDA-MB-231 cells treated with NPs (equivalent to DOX 10 μg/mL) or free DOX (10 μg/mL) suspended in RPMI-1640 medium at 37 °C for 4 h was scraped with a 200 μL pipette tip and observed with a light microscope after 24 h to evaluate the cell migration (proliferation) activity of tumor cells [33].

### 2.10. Statistical Evaluation

All data were evaluated using Student’s *t*-test or analysis of variance (ANOVA) at a significance level of *p* < 0.01 (**) [18,19,20,21,22,27,28,29,30,31,32].

## 3. Results and Discussion

### 3.1. Synthesis of DOX-Loaded Fe-Based HA NPs

To fabricate biofunctional DOX@aHA-DMA/Fe NPs, we first synthesized aHA-DMA using a biocompatible HA and a pH-responsive DMA. Briefly, aHA-DMA was synthesized by coupling DMA to aHA (obtained after the amination process of HA using ADH, DCC, NHS, and TEA) [10,22], as shown in Figure S1. As a result, we prepared two types of aHA-DMA with different DMA conjugation ratios (aHA-DMA_0.36_ and aHA-DMA_0.60_). Here, the molar conjugation ratio of DMA to aHA was defined as the number of conjugated DMA molecules per repeating unit of aHA and calculated after analyzing the ^1^H-NMR peaks at δ 2.4 ppm (-CH_3_, HA part) and δ 1.9 ppm (-CH_3_, DMA part) (Figures S2 and S3) [10,14,22]. Additionally, to determine the acidic pH-induced degradation of DMA moieties, we incubated aHA-DMA in an acidic PBS (150 mM, pH 6.8) environment for 24 h at 37 °C. We observed the disappearance of the ^1^H-NMR peaks at δ 1.9 ppm (-CH_3_, DMA part) (Figure S4) as a result of DMA degradation at pH 6.8 [14].

Next, we prepared three types of DOX-loaded Fe-based HA NPs (DOX@HA/Fe NPs, DOX@aHA-DMA_0.36_/Fe NPs, and DOX@aHA-DMA_0.60_/Fe NPs) after electrostatic interactions [4,5,12] between polymers (HA, aHA-DMA_0.36_, and aHA-DMA_0.60_), Fe^2+^, and DOX. The weight percentages (wt.%) of Fe^2+^ in DOX@HA/Fe NPs, DOX@aHA-DMA_0.36_/Fe NPs, and DOX@aHA-DMA_0.60_/Fe NPs were 0.63 ± 0.02 wt.%, 0.84 ± 0.03 wt.%, and 0.93 ± 0.02 wt.%, respectively. The loading efficiencies of DOX in DOX@HA/Fe NPs, DOX@aHA-DMA_0.36_/Fe NPs, and DOX@aHA-DMA_0.60_/Fe NPs were 65 wt.%, 63 wt.%, and 63 wt.%, respectively. The loading contents of DOX in DOX@HA/Fe NPs, DOX@aHA-DMA_0.36_/Fe NPs, and DOX@aHA-DMA_0.60_/Fe NPs were 20 wt.%, 20 wt.%, and 21 wt.%, respectively (Figure S5).

### 3.2. Characterization of DOX-Loaded Fe-Based HA NPs

We anticipated that the electrostatically complexed DOX@aHA-DMA/Fe NPs could have improved tumor uptake through binding CD44 receptors [11,15,17,18] of tumor cells due to the presence of HA and the accelerated DOX release rate by removal of DMA (i.e., pH-responsive property) at weakly acidic endosomal pH (i.e., pH 6.8) [11,12,14] (Figure 1a). First, we analyzed the pH-responsive property of DOX@aHA-DMA/Fe NPs using an FE-SEM instrument. Figure 1b shows that DOX@aHA-DMA_0.36_/Fe NPs and DOX@aHA-DMA_0.60_/Fe NPs became unstable at pH 6.8, and their nanostructures were collapsed probably due to ionic repulsion between Fe^2+^ and DMA-detached aHA (Figure S4). However, DOX@HA/Fe NPs (used as the control group) maintained no difference in nanostructured morphology regardless of the pH change. Similarly, Figure 1c shows that the average particle sizes of DOX@HA/Fe NPs, DOX@aHA-DMA_0.36_/Fe NPs, and DOX@aHA-DMA_0.60_/Fe NPs at pH 7.4 were 128 nm, 140 nm, and 144 nm, respectively. However, the average particle sizes of DOX@HA/Fe NPs, DOX@aHA-DMA_0.36_/Fe NPs, and DOX@aHA-DMA_0.60_/Fe NPs at pH 6.8 were shifted to 142 nm, 113 nm, and 52 nm, respectively. As expected, it was apparent that DOX@aHA-DMA_0.60_/Fe NPs with a high DMA conjugation ratio appears to have more reactivity to pH 6.8. In addition, the zeta potential values of DOX@HA/Fe NPs, DOX@aHA-DMA_0.36_/Fe NPs, and DOX@aHA-DMA_0.60_/Fe NPs at pH 7.4 were −8.2 mV, −7.6 mV, and −7.0 mV, respectively (Figure 1d). However, the zeta potential values of DOX@HA/Fe NPs, DOX@aHA-DMA_0.36_/Fe NPs, and DOX@aHA-DMA_0.60_/Fe NPs at pH 6.8 were shifted to −7.2 mV, −4.3 mV, and −0.5 mV, respectively. These results reveal that the degradation of DMA moieties in DOX@aHA-DMA_0.36_/Fe NPs and DOX@aHA-DMA_0.60_/Fe NPs at pH 6.8 promoted the electrostatic repulsion between aHA and Fe^2+^, resulting in mediating the destabilization of NPs.

In addition, all NPs showed no significant particle size change in PBS (150 mM, pH 7.4) containing 10% FBS for 7 days (data not shown).

### 3.3. In Vitro DOX Release

**Figure 2:**
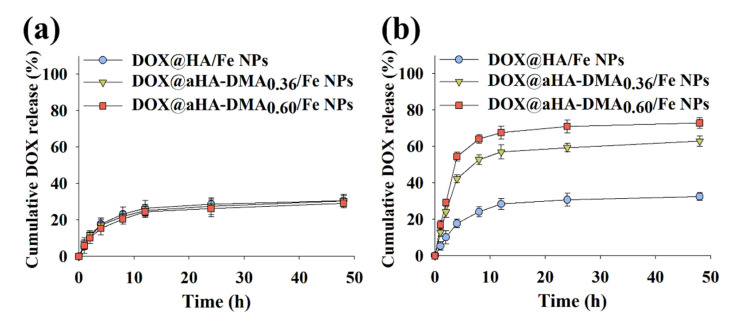
Cumulative DOX release profiles of DOX@HA/Fe NPs, DOX@aHA-DMA_0.36_/Fe NPs, and DOX@aHA-DMA_0.60_/Fe NPs at (**a**) pH 7.4 or (**b**) 6.8 for 48 h (*n* = 3, as multiple experiments).

We also monitored the DOX release behaviors of the DOX-loaded Fe-based HA NPs at pH 7.4 and 6.8. As shown in Figure 2, DOX-loaded Fe-based HA NPs showed a maximum 30 wt.% DOX release at pH 7.4, but it was confirmed that the cumulative DOX release of DOX@aHA-DMA_0.36_/Fe NPs and DOX@aHA-DMA_0.60_/Fe NPs increased at pH 6.8. In particular, DOX@aHA-DMA_0.36_/Fe NPs and DOX@aHA-DMA_0.60_/Fe NPs showed approximately 68 wt.% and 72 wt.% DOX release at pH 6.8, respectively. Here, the DOX release of DOX@aHA-DMA_0.36_/Fe NPs and DOX@aHA-DMA_0.60_/Fe NPs was rapid, reaching a plateau between 4 h and 12 h. These results indicate that DOX@aHA-DMA_0.36_/Fe NPs and DOX@aHA-DMA_0.60_/Fe NPs destabilized at pH 6.8 promote DOX release.

### 3.4. In Vitro Cellular Uptake of DOX-Loaded Fe-Based HA NPs

**Figure 3:**
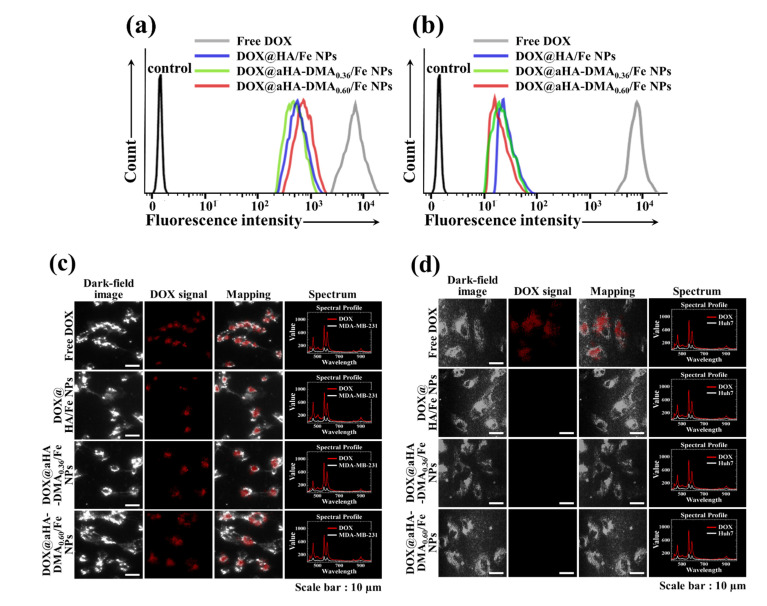
Flow cytometry analysis of (**a**) MDA-MB-231 cells and (**b**) Huh7 cells treated with free DOX (10 μg/mL) or NPs (equivalent to DOX 10 μg/mL) for 4 h of incubation at 37 °C. Here, control refers to cells that were not treated with drugs. Hyperspectral images of (**c**) MDA-MB-231 cells and (**d**) Huh7 cells treated with free DOX (10 μg/mL) or NPs (equivalent to DOX 10 μg/mL) for 4 h incubation at 37 °C.

To study the cellular internalization behaviors of the DOX-loaded Fe-based HA NPs, we performed in vitro cell tests using two types of tumor cells with or without CD44 receptors [11]. The quantitative cellular uptake of the DOX-loaded Fe-based HA NPs was measured using a flow cytometer. As shown in Figure 3a, the average fluorescent DOX intensity observed in MDA-MB-231 cells (with CD44 receptors) [11,31] was ~8.3 × 10^3^ (free DOX), ~7.9 × 10^2^ (DOX@HA/Fe NPs), ~8.1 × 10^2^ (DOX@aHA-DMA_0.36_/Fe NPs), and ~9.3 × 10^2^ (DOX@aHA-DMA_0.60_/Fe NPs). However, the DOX@HA/Fe NPs, DOX@aHA-DMA_0.36_/Fe NPs, and DOX@aHA-DMA_0.60_/Fe NPs showed low cellular internalization in Huh7 cells (without CD44 receptors) (Figure 3b) [11]. These results indicate that the DOX-loaded Fe-based HA NPs with HA moieties (DOX@HA/Fe NPs, DOX@aHA-DMA_0.36_/Fe NPs, and DOX@aHA-DMA_0.60_/Fe NPs) had excellent cellular uptake in MDA-MB-231 tumor cells with CD44 receptors due to HA-mediated binding to CD44 receptors. We further performed hyperspectral image analysis [11,17] to visualize the tumoral uptake of DOX-loaded Fe-based HA NPs by mapping the DOX signal spectrum in the cells. Figure 3c shows that MDA-MB-231 cells treated with DOX-loaded Fe-based HA NPs (DOX@HA/Fe NPs, DOX@aHA-DMA_0.36_/Fe NPs, and DOX@aHA-DMA_0.60_/Fe NPs) exhibited high cellular localization of DOX, unlike Huh7 cells (Figure 3d) treated with DOX-loaded Fe-based HA NPs. In addition, free DOX treatment showed high cellular localization regardless of the cell type [11].

### 3.5. In Vitro Tumor Inhibition of DOX-Loaded Fe-Based HA NPs

**Figure 4:**
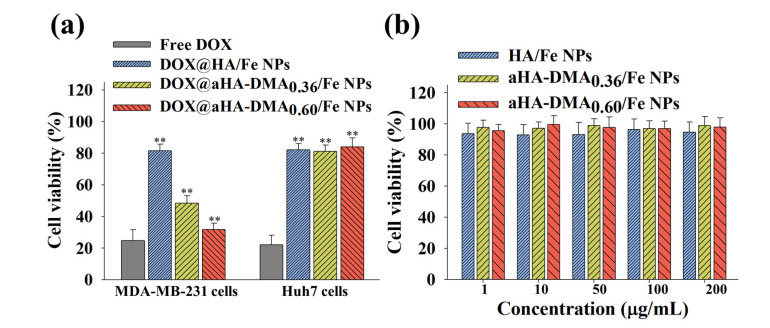
(**a**) Cell viability was determined by CCK-8 assay of MDA-MB-231 cells and Huh7 cells treated with free DOX (10 μg/mL) or NPs (equivalent to DOX of 10 μg/mL) for 24 h of incubation at 37 °C (*n* = 7, as multiple experiments, ** *p* < 0.01 compared to free DOX). (**b**) Cell viability was determined by CCK-8 assay of MDA-MB-231 cells treated with NPs (1–200 μg/mL, without DOX) for 24 h (*n* = 7, as multiple experiments).

To evaluate the in vitro tumor cytotoxicity of the DOX-loaded Fe-based HA NPs, we measured the cell viability of tumor cells treated with each sample [11,17,31]. Figure 4a shows that the DOX@aHA-DMA_0.60_/Fe NPs exhibited an excellent tumor cell death rate for MDA-MB-231 tumor cells compared with Huh7 cells. Interestingly, DOX@aHA-DMA_0.60_/Fe NPs resulted in an ~70% cell death rate for MDA-MB-231 tumor cells, unlike other HA-based NPs (DOX@HA/Fe NPs and DOX@aHA-DMA_0.36_/Fe NPs). This is thought to be due to the other properties (probably due to pH-dependent changes in NPs, Figure 2) in addition to the ability to bind CD44 receptors expressed in MDA-MB-231 tumor cells [11,17,31]. In addition, free DOX treatment resulted in high cytotoxicity to both cell lines. However, the NPs without DOX showed negligible cell cytotoxicity, indicating the non-toxic properties of NPs [11,17,31] (Figure 4b).

**Figure 5:**
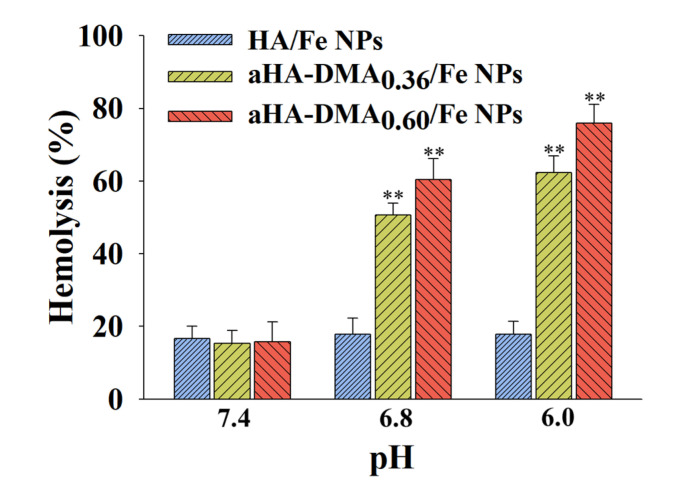
Hemolysis activity of the NPs (100 μg/mL, without DOX) at pH 7.4, 6.8, or 6.0 (*n* = 3, as multiple experiments, ** *p* < 0.01 compared to HA/Fe NPs).

Figure 5 shows the pH-dependent functionality of DOX@aHA-DMA_0.60_/Fe NPs in the cells. Here, we performed a hemolysis test [11,12,17,18] at pH 7.4, 6.8, or 6.0 using RBCs (as a model substance similar to the endosomal membrane) [11,12,17,18] to estimate the pH-dependent endosomolytic activity of NPs. First, negligible hemolytic activity of all NP samples at pH 7.4 was observed. However, the DOX@aHA-DMA_0.60_/Fe NPs resulted in remarkable hemolysis activity at pH 6.8 and 6.0, probably due to the proton sponge effect by the hydrolysis of DMA in aHA-DMA and the protonation of aHA at pH 6.8 and 6.0. In particular, the DOX@aHA-DMA_0.60_/Fe NPs with more DMA showed slightly better hemolysis activity than the DOX@aHA-DMA_0.36_/Fe NPs. This hemolysis activity of DOX@aHA-DMA_0.60_/Fe NPs is thought to mediate the endosomal escape of DOX released from NPs, resulting in improved cell cytotoxicity.

**Figure 6:**
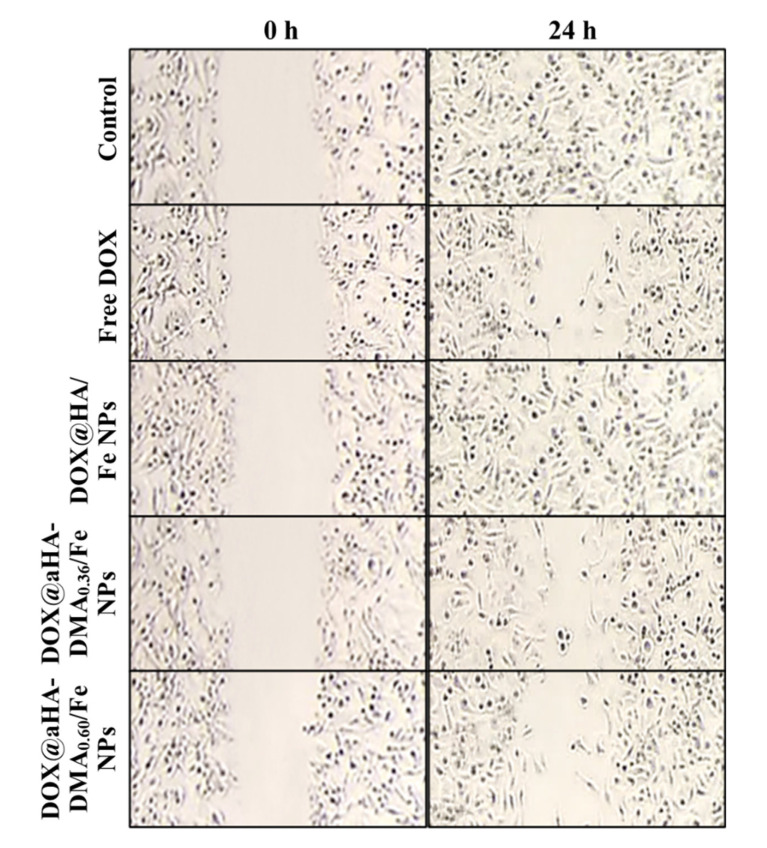
Local healing assays of MDA-MB-231 cells treated with free DOX (10 μg/mL) or NPs (equivalent to DOX 10 μg/mL) for 24 h of incubation at 37 °C.

We also investigated the therapeutic efficacy of DOX-loaded Fe-based HA NPs using a local healing test in MDA-MB-231 cells. As shown in Figure 6, MDA-MB-231 cells treated with DOX@aHA-DMA_0.60_/Fe NPs exhibited reduced tumor cell migration events [33] after 24 h of incubation, which is similar to the results of cells treated with free DOX. However, MDA-MB-231 cells treated with DOX@HA/Fe NPs presented some cell migration events, revealing their poor therapeutic efficacy [11,17,31,33].

Overall, our results suggest that the DOX released from DOX@aHA-DMA_0.60_/Fe NPs in an acidic endosomal environment (pH 6.0) is effective in inhibiting tumor cell proliferation and increasing tumor cell death.

## 4. Conclusions

In this study, we successfully engineered a metal-based multifunctional DOX@aHA-DMA_0.60_/Fe NPs to inhibit tumor cell proliferation. The electrostatically complexed DOX@aHA-DMA_0.60_/Fe NPs were selectively internalized to MDA-MB-231 tumor cells via CD44-mediated endocytosis. Importantly, DMA-detached aHA at acidic pH mediated ionic repulsion against Fe^2+^ ions in NPs and accelerated DOX release. The comprehensive results from in vitro studies indicate that DOX@aHA-DMA_0.60_/Fe NPs were effective in enhancing tumor cell suppression. Of course, further investigation is needed to confirm the antitumor activity of this formulation in vivo.

## Supplementary Materials

The following are available online, Figure S1: Synthesis procedure of aHA-DMA; Figure S2: ^1^H-NMR peaks of aHA; Figure S3: ^1^H-NMR peaks of aHA-DMA_0.60_; Figure S4: ^1^H-NMR peaks of DMA-detached aHA; Figure S5: DOX loading efficiency and loading content of DOX@HA/Fe NPs, DOX@aHA-DMA_0.36_/Fe NPs, and DOX@aHA-DMA_0.60_/Fe NPs (*n* = 3, as multiple experiments).

## Sample Availability

Samples of aHA-DMA and NPs are available from the authors.
