# Supplementary material for: Endosomal pH-Responsive Fe-Based Hyaluronate Nanoparticles for Doxorubicin Delivery
Source: Molecules. 2021 Jun 10;26(12):3547. doi: 10.3390/molecules26123547 (PMC8229704; doi:10.3390/molecules26123547)
Supplement: Supplementary file 1 [file molecules-26-03547-s001.zip › molecules-1248999-supplementary.pdf]

molecules

MDPI

Supplementary Data

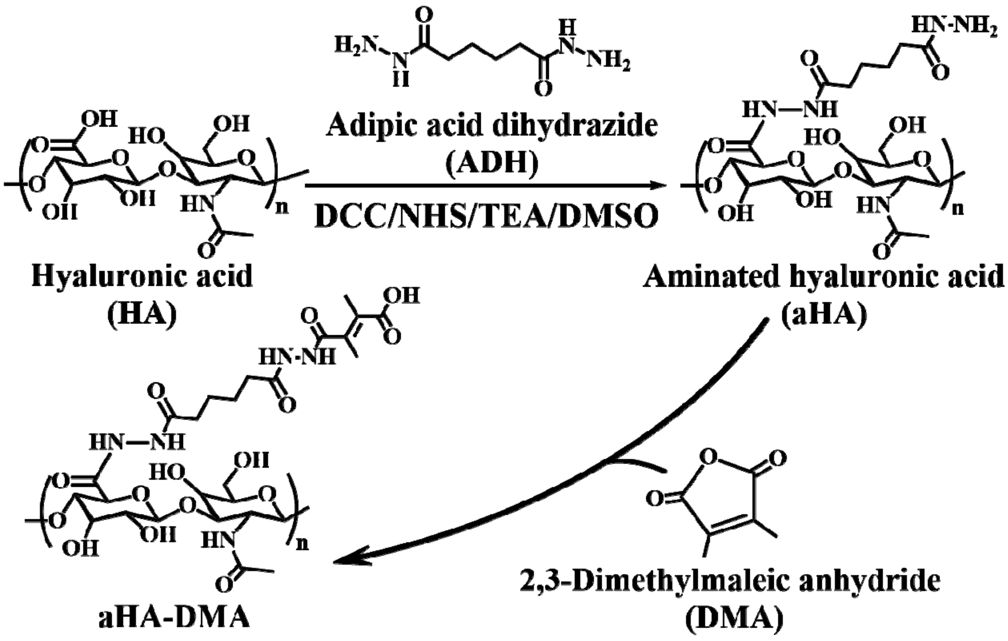

Figure S1. Synthesis procedure of aHA-DMA.

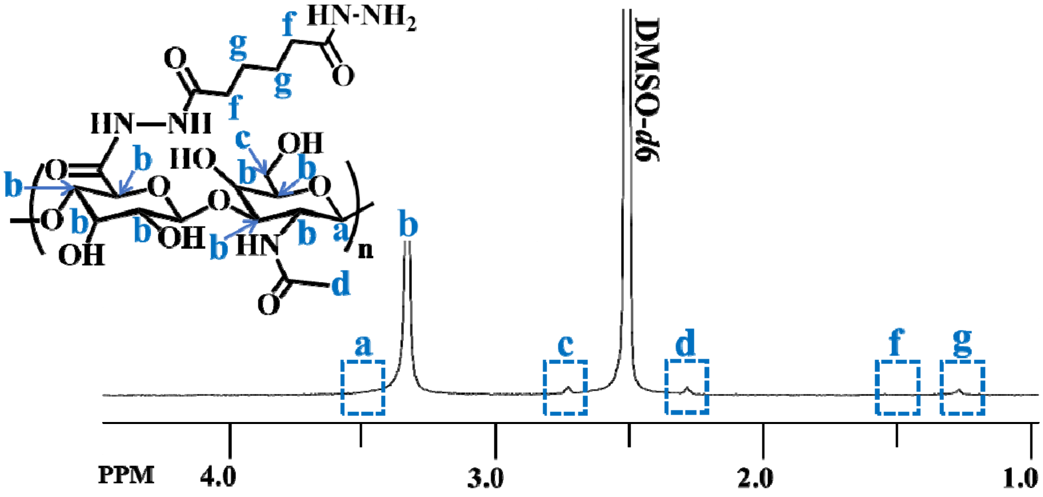

Figure S2. ^{1}H-NMR peaks of aHA.

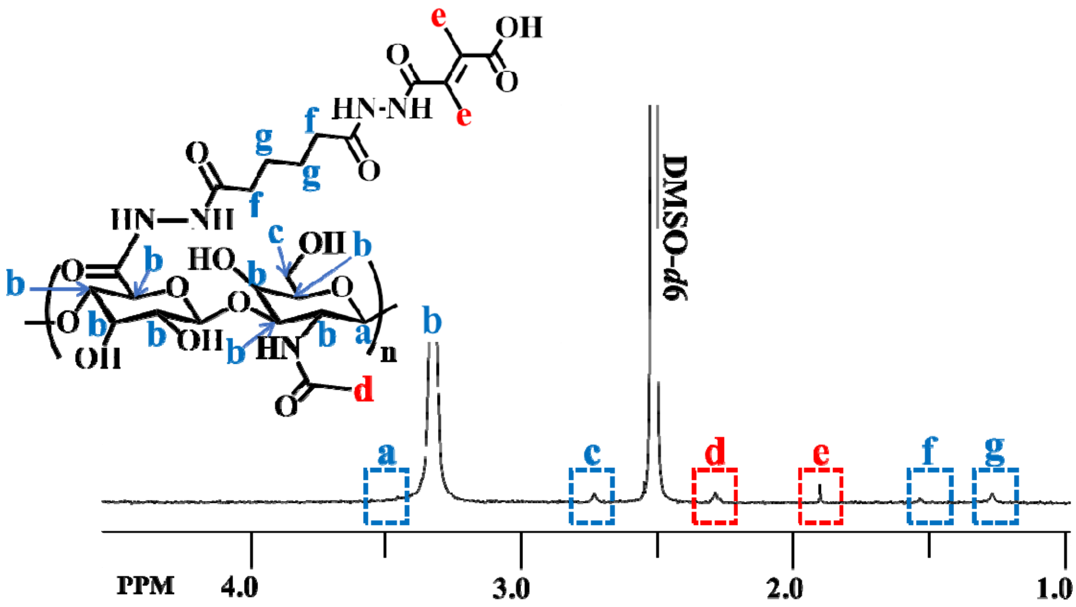

Figure S3. ^{1}H-NMR peaks of aHA-DMA$_{0.60}$.

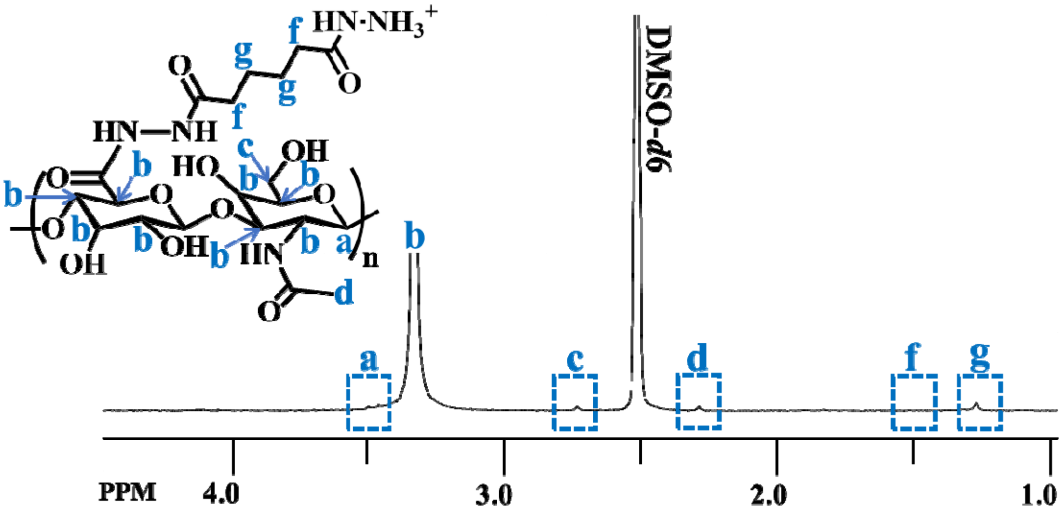

Figure S4. ^{1}H-NMR peaks of DMA-detached aHA.

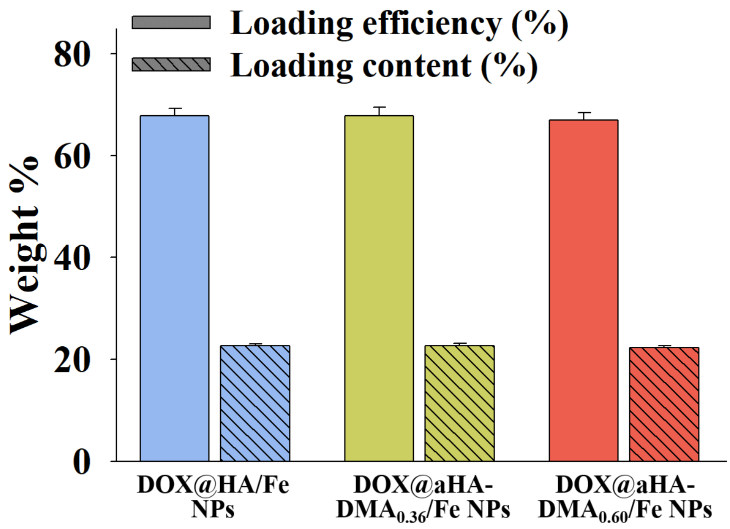

Figure S5. DOX loading efficiency and loading content of DOX@HA/Fe NPs, DOX@aHA-$DMA_{0.36}$/Fe NPs, and DOX@aHA-$DMA_{0.60}$/Fe NPs (n=3, as multiple experiments).
